# Physical Skill Training Increases the Number of Surviving New Cells in the Adult Hippocampus

**DOI:** 10.1371/journal.pone.0055850

**Published:** 2013-02-20

**Authors:** Daniel M. Curlik, Lisa Y. Maeng, Prateek R. Agarwal, Tracey J. Shors

**Affiliations:** Department of Psychology and Center for Collaborative Neuroscience, Rutgers University, Piscataway, New Jersey, United States of America; St. Jude Children’s Research Hospital, United States of America

## Abstract

The dentate gyrus is a major site of plasticity in the adult brain, giving rise to thousands of new neurons every day, through the process of adult neurogenesis. Although the majority of these cells die within two weeks of their birth, they can be rescued from death by various forms of learning. Successful acquisition of select types of associative and spatial memories increases the number of these cells that survive. Here, we investigated the possibility that an entirely different form of learning, physical skill learning, could rescue new hippocampal cells from death. To test this possibility, rats were trained with a physically-demanding and technically-difficult version of a rotarod procedure. Acquisition of the physical skill greatly increased the number of new hippocampal cells that survived. The number of surviving cells positively correlated with performance on the task. Only animals that successfully mastered the task retained the cells that would have otherwise died. Animals that failed to learn, and those that did not learn well did not retain any more cells than those that were untrained. Importantly, acute voluntary exercise in activity wheels did not increase the number of surviving cells. These data suggest that acquisition of a physical skill can increase the number of surviving hippocampal cells. Moreover, learning an easier version of the task did not increase cell survival. These results are consistent with previous reports revealing that learning only rescues new neurons from death when acquisition is sufficiently difficult to achieve. Finally, complete hippocampal lesions did not disrupt acquisition of this physical skill. Therefore, physical skill training that does not depend on the hippocampus can effectively increase the number of surviving cells in the adult hippocampus, the vast majority of which become mature neurons.

## Introduction

The dentate gyrus of the adult hippocampal formation generates thousands of new neurons every day. Although an estimated ten thousand new cells are born each day, the majority of these cells will die off within one-to-two weeks of their birth [1], [2]. However, if an animal engages in various forms of learning, that learning will rescue these cells from death, effectively increasing the number of new neurons that survive [1], [3]–[6]. The surviving neurons remain in the dentate gyrus for at least several months [5]. They integrate into the existing hippocampal circuitry [7], [8], and are presumably used during future learning experiences [9], [10].

Interestingly, only certain forms of learning rescue these cells from death. Learning an associative task in which the stimuli are separated from one another in time (trace conditioning) rescues the cells that are present and one week old at the time of training, whereas learning a similar task in which the stimuli overlap in time (delay conditioning) does not [1]. Likewise, only specific forms of spatial learning increase the number of cells that survive. Learning to navigate the hidden platform version of the Morris water maze rescues cells [1], [11], [12], whereas learning to locate a visible platform does not [1]. Together, these results raise a very important question regarding the relationship between learning and adult neurogenesis – Why do some, but not all, forms of learning rescue these new neurons from death?

Recent evidence suggests that learning preferentially rescues new neurons when that learning is difficult to achieve [4], [6]. This theory explains why trace, but not delay, conditioning rescues cells, as trace conditioning is considerably more difficult to learn, requiring many more trials to master [13]. In agreement with this theory, increasing the inter-stimulus interval increases the number of trials necessary to learn delay conditioning, and this type of learning is sufficient to increase the number of surviving neurons [14]. Conversely, decreasing the trace interval during trace conditioning decreases the number of trials necessary to learn, and this type of training does not increase the number of surviving neurons [15]. Our laboratory has repeatedly observed strong positive correlations between the number of trials an individual animal requires to learn a task, and the number of surviving cells in that animal’s dentate gyrus [4], [16], [17]. Thus, it appears that more effortful types of training are most effective in increasing neurogenesis via cell survival. Moreover, the cells only respond to training when the animal learns. Simply exposing an animal to the training apparatus and conditioning stimuli in a pseudorandom unpaired manner does not result in any change in cell number [1], [4]. Of those animals that are trained with trace conditioning, only those that successfully learn retain more of the new cells. Those that fail to learn, or that learn poorly, do not retain more cells than naïve animals [4], [11], [16], [18]. Together, these results suggest that successful learning rescues new neurons from death, but only when that learning is difficult to achieve [4], [6].

Based on these data, it was hypothesized that physical skill learning would rescue new hippocampal cells from death, provided that the skill was sufficiently difficult. Furthermore, we hypothesized that skill learning, but not simple physical activity, would rescue these cells. To test these hypotheses we trained groups of rats to acquire a difficult, or an easy, physical skill. We used a constantly accelerating rotarod procedure as our test of a difficult physical skill, and a slow constant velocity rotarod procedure as our test of an easy-to-perform skill. We chose the accelerating version of the rotarod because there is a strong learning component with the task [19]. Additionally, we investigated the effect of physical skill training on the survival of these cells as most forms of physical skill learning are believed to be hippocampal-independent [20], [21]. Instead, acquisition of these tasks is thought to rely on striatal, cerebellar, and cortical networks [22], [23]. However, hippocampal-independent processes of learning can still engage neurons within this structure [24]. For example, acquisition of the delay eyeblink response does not require an intact hippocampal formation [13], however this form of learning does increase the firing frequency of hippocampal neurons [25]. Moreover, one recent study has suggested that hippocampal-independent forms of learning may still increase the number of surviving adult-born hippocampal cells [18]. Therefore, we performed a third experiment to determine whether acquisition of our rotarod procedure required an intact hippocampal formation.

## Methods

### Subjects

Adult male Sprague-Dawley rats, ranging from 60 to 90 days of age were individually housed and given access to food and water *ad libitum*. Animals were maintained on a constant 12 hour light/dark cycle. The light cycle began at 7 a.m. and ended at 7 p.m. All procedures were designed to full comply with the PHS Policy on Humane Care and use of Laboratory Animals and the Guide for the Care and Use of Laboratory Animals. They were approved by the Rutgers University Animal Care and Facilities Committee (P.I. Tracey J. Shors, protocol nr. 98-018).

### Experimental Design

The study consisted of three separate experiments. In the first experiment, three groups of animals were used to investigate the effect of skill learning on the survival of adult-born hippocampal cells. All groups received one single intraperitoneal injection of 5-bromo-2-deoxyuridine (BrdU; 200 mg/kg) at the start of the experiment. BrdU is a marker of the S-phase of the cell cycle, and it labels cells currently dividing in the animal at the time of the injection, including the new neurons in the hippocampus. Seven days after the injection, when many of the new cells begin to undergo apoptosis, and when learning can rescue them from death [3], one group was trained with an accelerating version of the rotarod procedure. Previous research using associative and spatial learning has revealed that this is the optimal time to rescue these cells [3], [12]. If animals are trained immediately after BrdU-labeling there is no increase in cell survival [3]. Essentially, these cells express a critical period when they can be rescued from death, and that time period includes one-to-two weeks after they are created. Therefore, all animals began training one week after the BrdU injection.

**Figure 1:**
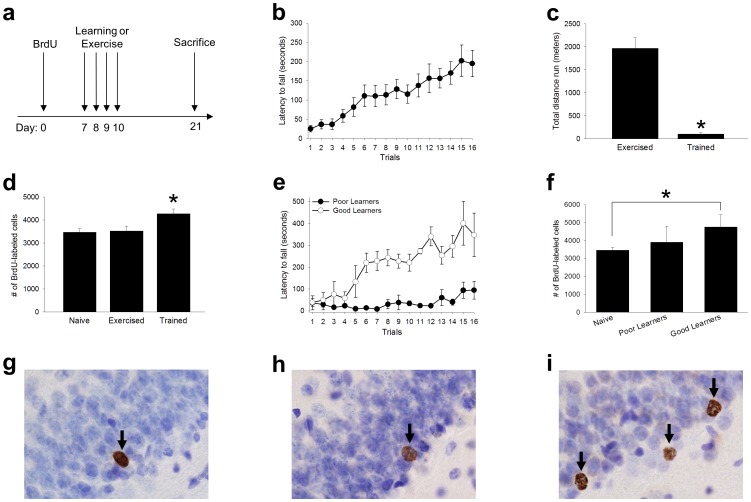
Physical skill training rescued new hippocampal cells from death. (A) Experimental design. Animals received one single intraperitoneal dose of BrdU (200 mg/kg), one week before training with the accelerating rotarod. Animals were trained with four trials per day, for four days. They were perfused twenty-one days after the BrdU injection. An additional group of animals were not trained. Instead, they were given access to activity wheels for four consecutive days, to examine the possibility that physical activity might rescue cells from death. (B) Trained animals increased their latency to fall from the rotarod. (C) The group given access to activity wheels (exercised) ran nearly twice as far as animals did during rotarod training (trained). (D) Acquisition of the physical skill rescued cells from death. The trained group retained more new cells than the naïve group, and the exercise group. Despite engaging in a large amount of physical activity the group given access to activity wheels did not retain any more cells than naïve animals. (E) Good learners consistently outperformed poor learners. (F) The good learners retained more new cells than naïve animals, whereas the poor learners did not. Representative BrdU-labeled cells from a (G) naïve, (H) exercise and (I) trained animal. Asterisks indicate *p≤*0.05. Arrows represent BrdU-labeled cells. Data represent mean ± s.e.m.

Rotarod training consisted of four trials per day, for four consecutive days. A second group of animals was not trained with the rotarod. Instead, one week after the BrdU injection, these animals were placed in new cages which allowed for free access to activity wheels. Following four days of exercise, these animals were returned to their home cages, which did not include activity wheels. A third group remained experimentally naïve. They were not exposed to the rotarod or the activity wheels. All animals were perfused twenty-one days after the BrdU injection, because cells that were not rescued from death would have undergone apoptosis at that time point. Therefore, the number of surviving BrdU-labeled cells at this common time point provided an index of how many new cells were rescued from death by the physical skill training and/or the exercise alone (Fig. 1a).

In a second experiment, we determined whether acquisition of an easier physical skill would rescue the new cells from death. Three groups were used in this experiment. One group was trained with the accelerating rotarod procedure. A second group was trained with a non-accelerating, slower velocity, version of the task. Both groups began training one week after the BrdU injection (200 mg/kg), and they were trained with four trials per day, for four days. The third group remained experimentally naïve.

The third experiment was designed to determine whether an intact hippocampus is necessary for the acquisition of the physical skill. The experiment consisted of two groups. One group received bilateral excitotoxic lesions of the entire hippocampal formation. These lesions were sufficient to eliminate both old and new neurons in the entire hippocampus. The second group received sham lesions. All animals were allowed at least one week to recover before training began. Animals were first trained with the accelerating rotarod procedure, with four trials per day, for four days. Following a three day break, all animals were then trained with the slow rotarod procedure, with four trials per day, for four days. Following training, all animals were perfused to determine the extent and location of the lesions, which were verified using the histological methods described below.

### Skill Training

The rotarod (Med Associates Inc. Model #ENV575) is a cylindrical rod that was elevated 26.7 cm above a platform. The rod was capable of accelerating, or maintaining a constant velocity, over a period of five minutes. Performance on a constantly accelerating version of the rotarod task has been reported to improve over several days of training. This increase in performance could not solely be accounted for by an increase in physical fitness, but rather the acquisition of a physical skill [19]. Thus, we chose the constantly accelerating rotarod procedure as our test of a difficult physical skill. Rats were placed on the rotarod, while it was stationary, facing away from the direction that the rod would begin rotating. In this position, the animal had to move forward to remain on the rod while it was rotating. Once all animals were placed on the stationary rod in the correct orientation a trial began.

During each trial with the accelerating procedure the rotarod linearly accelerated from 1.47 cm/sec to 14.74 cm/sec over a five-minute period. After five minutes the rod no longer accelerated, and it remained at the constant maximum velocity of 14.74 cm/sec. The behavioral measure was the latency to fall from the rod. Animals were allowed to remain on the rod until they fell off or until ten minutes had passed. The time from the start of one trial to the start of the next was twenty minutes. Training with the slow velocity version of the task was similar. However, during each trial the rotarod did not accelerate. Instead, it began and remained at the slow constant velocity of 2.94 cm/sec.

Animals trained with the accelerating rotarod procedure were later parsed into “good learners” and “poor learners” based on their behavioral performance. The animals were divided into four quartiles, based on their average latency to fall from the rotarod during all sixteen trials of training. Good learners were those in the top quartile, with the greatest average latency, whereas poor learners were those in the bottom quartile, with the smallest average latency. When group sizes were not evenly divisible by four, we rounded down.

### Procedure for Activity Wheels

Animals were removed from their home cages and placed into new cages, which allowed for free access to an attached activity wheel (Med Associates Inc. Model #ENV046). The activity wheels were 35.6 cm in diameter (111.78 cm circumference). The wheels were connected to an automated computer system, which allowed for continuous recording of the number of quarter rotations made by each animal every hour. Animals were placed in these new cages at 4:30pm, and they remained in them for eighty-eight hours, before being returned to their home cages at 8:30am, following four dark cycles in the new cages.

### Immunohistochemistry for BrdU

Twenty-one days after the BrdU injection all animals were deeply anesthetized with sodium pentobarbital (100 mg/kg) and transcardially perfused with 4% paraformaldehyde. Brains were extracted and post-fixed in 4% paraformaldehyde at 4 degrees Celsius for 24hours before being transferred to phosphate buffered saline (PBS). Forty-micrometer coronal sections were taken through the entire rostral-caudal extent of the dentate gyrus of one hemisphere, and every twelfth section was mounted onto a glass slide. Tissue was then stained for the presence of BrdU using standard peroxidase methods [4]. Briefly described, slides were pretreated by heating in 0.1 M citric acid (pH 6.0). Slides were then rinsed in PBS, incubated in trypsin for 10 minutes, denatured in 2N HCl for 30 minutes, rinsed and incubated overnight in primary anti-mouse BrdU (1∶200, Becton Dickson) and 0.5% Tween 20. The next day the slides were incubated in biotinylated anti-mouse antibody (1∶200, Vector Laboratories) for 60 minutes, before being placed in avidin-biotin complex (1∶100, Vector Laboratories) for sixty minutes. The slides were then placed in diaminobenzidine for four minutes, counterstained with cresyl violet, and coverslipped with Permount glue (Fischer Scientific, Fair Lawn, NJ). All slides were coded so that the researcher was blind to the experimental condition of each slide. The number of BrdU-positive cells in the dentate gyrus of each slice (granule cell layer+hilus) was counted by hand. All cell counts were multiplied by 24 (2 hemispheres X every 12th section), to estimate the total number of BrdU-labeled cells present in the entire dentate gyrus of both hemispheres. All imaging was performed on a Nikon Eclipse 80i light microscope with a DS-Fi1 Nikon camera, with the NIS Elements software.

### Hippocampal Lesion Surgery and Histology

All rats were anesthetized with sodium pentobarbital (60 mg/kg, i.p.). The scalp was cleaned with Betadine before incisions are made. Rats received either sham, or hippocampal, lesions. Sham animals received bilateral infusions of 0.1% phosphate buffered saline (PBS). Rats with lesions of the hippocampus were bilaterally infused with the excitotoxin N-methyl-D-asparate (NMDA) at a dose of 20 mg/ml. All infusions (volume: 0.35 µl; rate: 0.5 µl/minute) were made via a Stoelting microinfusion pump at 12 sites within the hippocampus (AP: –2.5 mm, ML: ±1.6, DV: –3.8 mm; AP: –4.2 mm, ML: ±2.6, DV: –3.1 mm; AP: –5.3 mm, ML: ±5.0 mm, DV: –5.9 mm; AP: –5.3 mm, ML: ±4.2 mm, DV: –3.4 mm; AP: –5.8 mm, ML: ±4.6 mm, DV: –6.1 mm; AP: –6.0 mm, ML: ±5.6 mm, DV: –4.1 mm). AP coordinates were measured relative to Bregma, ML coordinates from the midline, and DV coordinates from the surface of the brain (dura). Each infusion occurred two minutes after the insertion of the needle to the infusion site, to allow time for the tissue to settle. The infusions were separated from the end of one to the start of the next by five minutes. Following the completion of infusions, the holes in the skull were covered with bone wax. All animals were single-housed after surgery. They were allowed at least one week to recover before the start of behavioral testing.

After behavioral testing all rats were heavily anesthetized with a sodium pentobarbital solution (Sleepaway, Butler Schein) and perfused with 0.9% saline solution followed by 10% buffered formalin. Brains were removed and post-fixed in 10% formalin for at least 24 h. They were then transferred to a 30% sucrose-formalin cryoprotectant solution, where they were allowed at least three days to become fully saturated with the sucrose-formalin. The brains were then sectioned into fifty micrometer coronal sections using a cryostat. The sections were mounted onto gelled slides and stained with 0.1% cresyl violet to verify the lesions. Rats were excluded from the study if the lesions were misplaced or incomplete. Lesions were identified by the location of the needle track, absence of nerve cell bodies, gliosis, or the presence of darkly stained astrocytes [26]. Animals, both sham and lesioned, were also excluded if there was extensive damage to extra-hippocampal areas, i.e. tissue damage due to needle placement.

## Results

In the first experiment, we determined how well the animals learned the accelerating rotarod procedure, and whether this training increased the number of remaining BrdU-labeled cells. One group of rats was trained with an accelerating rotarod procedure (Trained, *N* = 13). Repeated measures analysis of variance indicated that this group increased their latency to fall from the rotarod as training progressed (*F*_15,180_ = 8.96, *p*≤0.01; Fig. 1b). A second group was not trained with the rotarod. Instead, they were placed in new home cages, which allowed for free access to activity wheels for four consecutive days (Exercised, *N* = 15). A third group remained experimentally naïve (Naïve, *N* = 18). Animals given access to activity wheels ran almost twenty times farther than animals did during rotarod training (*t*_26_ = 7.56, *p*≤0.01; Fig. 1c). The number of surviving BrdU-labeled cells differed between these three groups (*F*_2,43_ = 4.96, *p*≤0.05; Fig. 1d, g-i). Post-hoc Tukey comparisons revealed that the trained group retained more new cells then both the naïve group (p≤0.05), and the exercise group (p≤0.05). Despite engaging in a large amount of physical activity, the group that exercised in the wheels did not retain any more new cells than naïve animals (p>0.05). These results suggest that skill learning, and not merely physical exercise, rescued hippocampal cells from death (Fig. 1d).

Previous studies indicate that learning only rescues cells from death when that learning is successful [4], [18]. To examine this hypothesis in the current study we separated trained animals into good learners (*n* = 3) and poor learners (*n* = 3), based on their behavioral performance. Repeated measures analysis of variance of these two conditions, with trial as the repeated measure, revealed main effects of trial (*F*_15,60_ = 7.02, *p*≤0.01), and learning condition (*F*_1,4_ = 28.87, *p*≤0.01), and a significant trial by condition interaction (*F*_15,60_ = 4.2, *p*≤0.05), indicating that the good learners increased their performance over the four days of training, whereas the poor learners did not (Fig. 1e). A difference in the number of surviving cells was observed between the good learners, poor learners, and naïve animals (*F*_2,21_ = 4.86, *p*≤0.05). Post-hoc Tukey comparisons indicated that the good learners retained more new cells than the naïve animals (p≤0.05), whereas the poor learners did not (p>0.05; Fig. 1f). Together, these results reveal that the acquisition of a physical skill can increase the number of surviving cells in the adult dentate gyrus.

**Figure 2:**
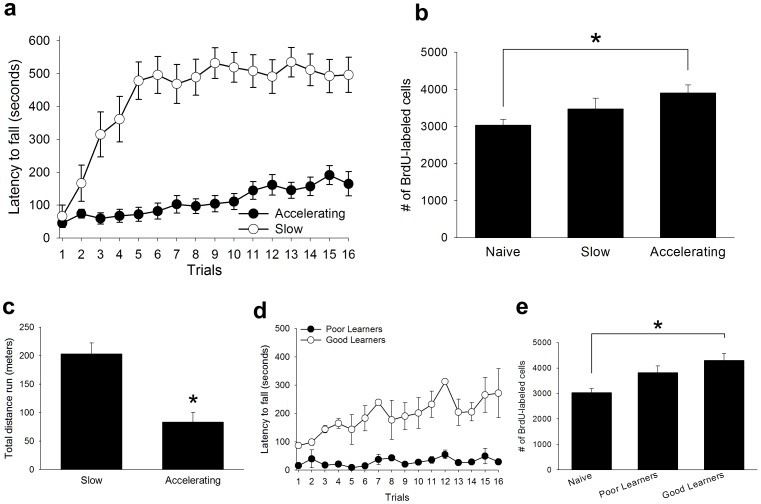
Training with the accelerating, but not the slow, rotarod procedure rescued cells. (A) Both trained groups increased their latency to fall from the rotarod. However, the group trained with the slow procedure outperformed the group trained with the accelerating procedure. (B) The group trained with the accelerating task retained more new cells than naïve animals, whereas the group trained with the slow task did not. (C) Animals ran twice as far during training with the slow procedure than during training with the accelerating procedure. (D) Good learners outperformed the poor learners during accelerating training, (E) and the good learners retained more new cells than animals that were not trained. Poor learners displayed no such increase in cell survival. Data represent mean ± s.e.m.

It has been theorized that learning will only rescue cells from death when the learning is difficult to achieve, requiring many trials to master [4], [6]. To determine whether acquisition of a difficult, but not an easy, physical skill rescues cells from death, we trained one group with the accelerating rotarod procedure (Accelerating, *N* = 18). A second group was trained with a slow version of the task (Slow, *N* = 17). In this case, the rotarod started at a slow constant velocity and it remained at that velocity for the duration of the trial. By removing the acceleration we predicted that this slow procedure would be easier to master than the accelerating procedure. Repeated measures analysis of variance revealed a main effect of trial (*F*_15,495_ = 19.383, *p*≤0.01), and training conditioning (*F*_1,33_ = 62.20, *p*≤0.01), with a significant trial by condition interaction (*F*_15,495_ = 9.66, *p*≤0.01), indicating that the group trained with the slow procedure had a greater latency to fall from the rotarod than the group trained with the accelerating procedure (Fig. 2a). Separate repeated measures ANOVAs conducted for each training condition revealed a main effect of trial for both the accelerating (*F*_15,255_ = 5.37, *p*≤0.01), and the slow (*F*_15,240_ = 15.43, *p*≤0.01) conditions. These results reveal that both groups increased their latency to fall from the rod as training progressed. The group trained with the slow rotarod procedure also travelled twice as far as the group trained with the accelerating procedure (*t*_33_ = 4.65, *p*≤0.01; Fig. 2c).

The number of surviving BrdU-labeled cells was assessed using a univariate analysis of variance with the training condition as the independent measure, and the number of surviving BrdU+ cells as the dependent measure. There was a significant main effect of training condition (F_2,63_ = 4.72, p≤0.05). Post-hoc Tukey comparisons revealed that the group trained with the accelerating rotarod procedure retained more BrdU-labeled cells than naïve animals (p≤0.05), whereas the group trained with the slow speed procedure did not (Fig. 2b; p>0.05). Rats in the accelerating condition were separated into good learners (*n* = 4) and poor learners (*n* = 4). Repeated measures analysis of variance revealed a significant effect of trial (*F*_15,90_ = 3.67, *p*≤0.01), and learning condition (*F*_1,6_ = 649.46, *p*≤0.01), with a significant trial by condition interaction (*F*_15,90_ = 2.96, *p*≤0.01; Fig. 2d). The number of BrdU-labeled cells differed between the good learners, poor learners, and naïve animals (*F*_2,20_ = 4.34, *p*≤0.05). Good learners retained more new cells than naïve animals (p≤0.05), whereas the poor learners did not (p>0.05; Fig. 2e).

**Figure 3:**
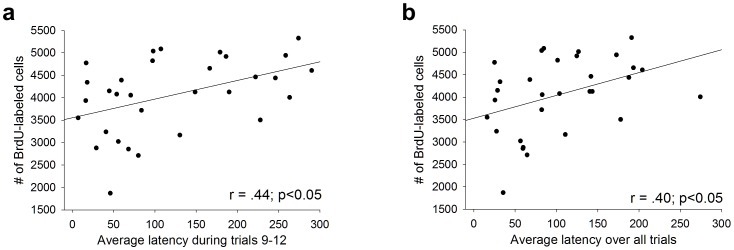
Animals that mastered the task retained more new cells. (A) The average performance on the third day of training and (B) the average performance across all four days of training significantly correlated with the number of surviving adult-born hippocampal cells.

In the first experiment, there was a positive correlation between the average latency to fall during the third day of training and the number of surviving cells (r = 0.56, p≤0.05). The average latency across all four days of training (r = 0.35, p>0.05), or each animal’s greatest latency to fall from the rod during any one trial (r = 0.20, p>0.05) did not significantly correlate with the number of surviving cells. In the second experiment, no significant correlations were observed between behavioral performance and cell survival. The average latency to fall during the third day of training (r = 0.38, p>0.05), the average latency across all four days of training (r = 0.42, p>0.05), and the greatest latency to fall during any one trial (r = 0.45, p>0.05), did not significantly correlate with the number of surviving cells. However, across both experiments all of these correlations were in the positive direction. Therefore, we combined the data from all of the animals that were trained with the accelerating procedure and examined correlations between behavioral performance and cell survival. In this relatively large sample (*N* = 31), the average latency on the third day of training (r = .44, p≤0.05; Fig 3a), the average latency across all four days of training (r = .40, p≤0.05; Fig. 3b), and the greatest latency during any one trial (r = .36, p≤0.05) were all significantly correlated with the number of BrdU-labeled cells.

**Figure 4:**
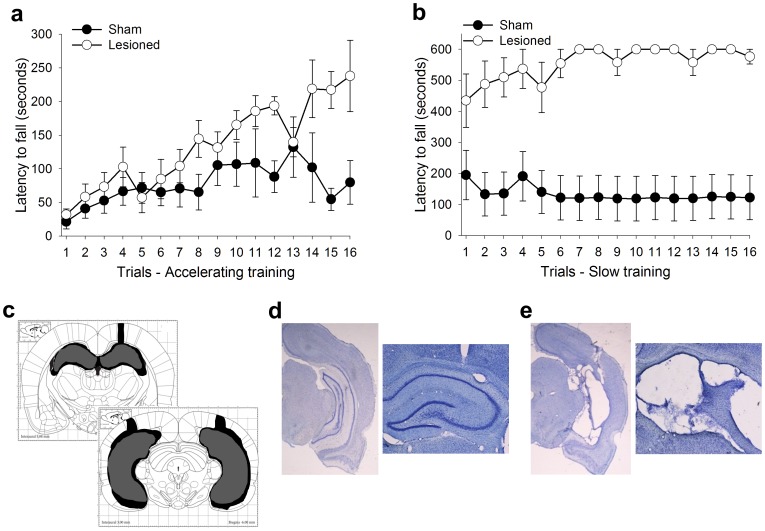
Hippocampal lesions did not impair skill acquisition. (A) Subjects with and without a functional hippocampus successfully learned the accelerating rotarod procedure. (B) During training with the slower version of the task the lesioned animals outperformed the sham animals. (C) Reconstructions of largest (black) and smallest (gray) lesions [41]. Histology from a representative (D) sham-operated animal and (E) a hippocampal lesioned animal. Data represent mean ± s.e.m.

In a third experiment, bilateral excitotoxic lesions of the entire hippocampal formation (Lesion, *N* = 9) or sham lesions (Sham, *N* = 19) were performed in new groups of animals before training with the accelerating rotarod procedure (Fig. 4c–e). Repeated measures analysis of variance, with trial as the repeated measure, and lesion condition as the independent measure, revealed a significant effect of trial (*F*_15,405_ = 10.84, *p*≤0.01 ), no effect of lesion condition (*F*_1,26_ = 2.27, *p*>0.05), and a trial by lesion condition interaction (*F*_15,390_ = 2.42, *p*≤0.01). Separate repeated measures ANOVAs conducted for each group revealed that both the sham (*F*_15,270_ = 4.37, *p*≤0.01), and the lesioned (*F*_15,120_ = 7.64, *p*≤0.01) groups increased their latency to fall from the rotarod during training (Fig. 4a), indicating that both groups acquired the task. Following training with the accelerating procedure both groups were then trained with the slow procedure. The latency to fall from the rotarod during slow training was examined using a repeated measures ANOVA with trial as the repeated measure, lesion condition as the independent measure, and latency to fall as the dependent measure. This analysis revealed no main effect of trial (*F*_15,390_ = 0.66, *p*>0.05), but a significant effect of lesion condition (*F*_1,26_ = 16.04, *p*≤0.01) and a trial by lesion condition interaction (*F*_15,390_ = 4.36, *p*≤0.01), indicating that the lesioned group outperformed the sham group during this slow procedure (Fig. 4b). Together, these results reveal that an intact hippocampal formation is not necessary for acquisition of the physical skills necessary to perform the accelerating or the slow rotarod procedures.

Animals without an intact hippocampus did not fully maintain performance between the end of each daily session and the beginning of the next. In other words, the lesioned animals fell off the rotarod sooner on the first trial of training on each day. For example, animals with complete hippocampal lesions fell off the rod in about 103 seconds on the last trial of the first day of training, but fell off in about 57 seconds during the first trial of the next day. This change was not observed in the sham-lesioned group, or in any of the groups trained in the first two experiments, which did not receive any form of surgery (Fig. 4a). During training with the slow procedure, lesioned animals also displayed this decrease in performance on the first trial of each day, although it was not as pronounced as during the accelerated rotarod test. For example, animals in the lesioned group fell off the rod in about 537 seconds during the last trial of day one but fell off in 477 seconds on the first trial of the next day. Again, a similar between sessions decrease in performance was not observed in sham animals (Fig. 4b).

Next, we assessed whether acquisition of the accelerating version of the rotarod procedure occurred between or within training sessions, in animals without lesions. To do this, a repeated measures analysis of variance was performed, with trial as the repeated measure, session number as the independent measure, and latency to fall as the dependent measure. This analysis revealed a significant effect of session (*F*_3,72_ = 3.05, *p*≤0.05), with no effect of trial (*F*_3,216_ = 0.79, *p*>0.05), and no trial by session interaction (*F*_9,216_ = 1.29, *p*>0.05). These results indicate that animals increased their latency to fall off the rod between sessions, but not within sessions (There were no differences within each daily session). This same analysis was then performed using data from the lesion group. In this case, there were main effects of session (*F*_3,32_ = 8.66, *p*≤0.01), and trial (*F*_3,96_ = 11.24, *p*≤0.01), with no session by trial interaction (*F*_9,96_ = 0.37, *p*>0.05). Therefore, these data indicate that animals without an intact hippocampus increased their performance both within and between each daily session.

Next, we assessed these same measures for the slow procedure. A repeated measures ANOVA was conducted for animals in the sham condition during slow training, with trial as the repeated measure, session number as the independent measure, and latency to fall as the dependent measure. This analysis indicated a significant effect of session (*F*_3,72_ = 3.05, *p*≤0.05), with no effect of trial (*F*_3,216_ = 0.79, *p*>0.05), and no trial by session interaction (*F*_9,216_ = 1.29, *p*>0.05). Interestingly, this main effect of session reflected a decrease in latency to fall from the rotarod across sessions, perhaps as a result of overtraining. This same analysis was then conducted using data from the lesion group. This revealed main effect of trial (*F*_3,96_ = 11.24, *p*≤0.01), and session (*F*_3,32_ = 8.70, *p*≤0.01), with no trial by session interaction (*F*_9,96_ = 0.37, *p*>0.05). Overall, these results confirm that the hippocampal formation is not necessary for learning the motor skills associated with either the accelerating version or the slow speed version of this test.

## Discussion

The data presented here indicate that physical skill training can rescue new hippocampal cells from death. These cells are located in the granule cell layer of the hippocampus, where ∼80% of the surviving cells differentiate into granule neurons [5], [16]. The task that was most effective involved learning to maintain balance on a rod that was turning and accelerating in speed. Animals trained with this task retained more new cells than those that were not trained. However, those that learned the task retained more new cells than those that did not learn well. These data are consistent with correlations between individual performance and the number of surviving cells. Interestingly, the cells were not preferentially retained in animals that were trained on a simpler task, in which the rod rotated very slowly. Thus, it would appear that training on a more challenging physical task is more effective in keeping new neurons alive than training on a less challenging task. Exactly why the more difficult task is effective is not known. It is not because it is hippocampal dependent – because it is not. In fact, animals without a hippocampus learned well and even outperformed their sham-lesioned counterparts. This increase in performance may have arose because hippocampal lesions are associated with increased motor activity [27], [28]. However, the important point is that animals could learn the skill without a hippocampus. Therefore, acquisition of a physical motor skill that does not depend on the hippocampal formation for learning is nonetheless sufficient to increase the survival of new cells that are already present within the hippocampus at the time of training.

Voluntary exercise did not increase cell survival. Animals given access to activity wheels ran nearly twenty times farther than animals that were trained with the rotarod test, yet they did not retain significantly more cells than animals that were sedentary in their home cages. It is important to note that we assessed the cells that were already present at the time of the exercise. Many studies report that aerobic exercise increases the number of new neurons in the dentate gyrus [29], but most of these studies investigate cell proliferation, or do not intentionally distinguish between effects of exercise on cell proliferation versus effects of exercise on the survival of cells that are already present. Overall, exercise seems to exert its’ greatest effects on cellular proliferation, whereas learning tends to influence the number that survive. However, there is a report that two weeks of exercise can increase the survival of cells that are already present during the activity [30]. Therefore, it is possible that prolonged periods of physical activity increase neuronal survival, whereas acute periods do not. Nonetheless, in the present study, there was no observable increase in cell survival in response to physical exercise, whereas there was a significant increase in cell survival in response to physical skill training. That said, we did not assess heart rate or oxygen intake during rotarod training or exercise. It is possible that training with the accelerating rotarod increases the number of surviving cells because it is more intense and/or aerobic than running in the wheels alone. Future research is needed to explore this possibility. Regardless, the present findings suggest that physical skill training and not exercise alone increases the number of surviving cells in the adult hippocampus. Because the physical skill training began when the new neurons were about to undergo apoptosis it is presumed that training rescued them from death [3].

Training with the rotarod procedure increased cell survival even though the hippocampus was not necessary for acquisition of the skill. This kind of effect is not unprecedented, as forms of learning that do not require the hippocampus can alter activity within this region [13], [25]. Moreover, hippocampal activity can alter non-hippocampal dependent forms of learning. For example, ablation of the hippocampal formation does not disrupt acquisition of the delay eyeblink response [13]. However, the systemic administration of scopolamine does impair this learning. Interestingly, scopolamine only impairs delay conditioning in animals that have an intact hippocampus. Animals without an intact hippocampus readily learn the delay eyeblink response in the presence of, or without, scopolamine [31]. These results indicate that hippocampal activity can modulate forms of learning that do not require the hippocampus. In the present study, hippocampal activity may be altered in such a way as to influence the survival of the adult-born cells, but we do not know the mechanism. It is noted that animals without an intact hippocampus consistently decreased their performance during the first trial of each day of training (Fig. 4a, 4b). These data suggest that there was less consolidation from one day to the next in animals without a hippocampus, and they are in agreement with one recent study which reveals that the hippocampus may contribute to the consolidation of motor sequence learning in humans [32]. Therefore, neuronal mechanisms which contribute to consolidation processes involved in motor skill acquisition may selectively affect the new cells. Minimally, our current data indicate that new cells within the hippocampus are engaged by this form of physical skill training, because their survival is increased by the training experience.

It is also possible that neuronal activity in regions outside of the hippocampus may modulate hippocampal activity during rotarod training. Acquisition of many motor skills, including those associated with the rotarod procedure, requires an intact cerebellum [33], and changes to cerebellar activity can alter motor performance. For example, the activity of H2 receptors in the cerebellar fastigial nucleus modulates performance during rotarod training [34]. Because neuronal activity within the fastigial nucleus modulates, and is modulated by, activity within the hippocampal formation [35], cerebellar activity during the rotarod training may have influenced the number of new hippocampal neurons that survived, by altering neuronal activity within the hippocampus. Of course, this is only one possible explanation for how this form of physical skill training rescues new neurons from death. We hope that the present results and procedures provide other laboratories with an easy, and straightforward, method of manipulating hippocampal cell survival through rotarod training, so that these and other related questions can be pursued.

It is important to note that animals that learn better do not begin training with more cells than those that fail to learn. Rather, these good learners retain more of the cells that are already present, and they do so in response to the training experience [3], [36], [37]. For example, in one study, animals were injected with BrdU just as training began. This time point was chosen to assess how many cells the animals possessed and/or produced during training. There was no increase in cell number in the trained animals, which suggests that training does not increase the number of cells that the animals produce either before or during training [3]. Moreover, there was no correlation whatsoever between performance on the task and cell number [37]. Additionally, we have trained animals to determine their learning ability and then labeled new cells weeks later (cells that were not yet present during the initial training). The animals were then retrained on the same task. Those that had learned well before we labeled the new cells did not produce or retain more cells than naïve animals. Because the group of naïve animals would presumably include both good learners and poor learners, these data suggest that those animals that learn well do not produce more cells than those that learn poorly. Furthermore, animals trained with delay conditioning followed by trace conditioning did not retain any more cells than the naïve animals, despite the fact that they learned well [14]. We have published several other studies during which animals learn well – again with no increase in cell number when compared to the naïve controls or animals trained with unpaired stimuli [14], [15], [17]. These results suggest that the intrinsic number of cells does not necessarily predict how well an animal will learn. That said, all of the aforementioned studies were conducted using associative classical eyeblink conditioning tasks, and therefore it is possible that animals that learn a rotarod test better may produce more cells that live longer. But given the plethora of evidence using other training procedures, it does seem unlikely.

Several studies have reported that new neurons in the hippocampus are necessary for various forms of learning and memory. For example, ablating neurogenesis disrupts trace eyeblink conditioning [10] and trace fear conditioning [38]. However, not all forms of learning are disrupted. For example, similar manipulations do not disrupt delay eyeblink conditioning or spatial learning [1]. Furthermore, the present data indicate that new (or old) neurons are not required for performing the physical skill used here. We have proposed that these new neurons are rescued from death to prepare the brain for future learning experiences [6]. In support of this hypothesis, one recent study trained animals on two learning tasks, both of which increase cell survival [37]. Training on the one task enhanced acquisition during training on the second task. As a consequence, cells that were born after the first training experience were more likely to survive. These findings suggest a mutually beneficial relationship (perhaps a positive feedback system) between learning and neurogenesis, which enhances future learning and the number of surviving neurons in the adult brain.

Post-mortem studies confirm that adult-born neurons are generated in the human hippocampal formation [39], [40]. However, it is not yet possible to assess the effects of learning on neurogenesis and cell survival in humans. In principle, learning a cognitively-demanding physical skill, such as a new dance or sport, should increase the number of cells that survive to become mature neurons in the human hippocampus.
